# Assessing Traditional Chinese Medicines for Anti‐Dengue Using a National Health Insurance Research Database and Bioassays

**DOI:** 10.1002/fsn3.70009

**Published:** 2025-02-28

**Authors:** Wan‐Yu Su, Hsiang‐Hui Lo, Yuan‐Liang Wen, Jeng‐Wei Lu, I‐Chuan Yen, Yi‐Jung Ho, Li‐Ting Kao

**Affiliations:** ^1^ School of Pharmacy National Defense Medical Center Taipei Taiwan, ROC; ^2^ Department of Pharmacy Tri‐Service General Hospital Taipei Taiwan, ROC; ^3^ Graduate Institute of Life Sciences National Defense Medical Center Taipei Taiwan, ROC; ^4^ Biotech Research and Innovation Centre University of Copenhagen Copenhagen Denmark; ^5^ The Finsen Laboratory Rigshospitalet/National University Hospital Copenhagen Denmark; ^6^ Department of Bioscience and Biotechnology National Taiwan Ocean University Keelung Taiwan, ROC

**Keywords:** dengue fever, dengue virus, National Health Insurance Database, traditional Chinese medicine

## Abstract

Dengue fever is a widespread viral infectious disease transmitted by mosquitoes. Despite the potentially fatal consequences for some individuals, no antiviral drugs have been approved for the treatment of this disease. Taiwan's National Health Insurance Database (NHID) holds extensive medical records for almost every resident, encompassing conventional and traditional Chinese medicine (TCM) treatments. This study aims to uncover potential TCM formulas with antiviral properties against dengue fever by analyzing NHID data. Initially, the NHID was screened to identify candidate TCM formulas. Subsequently, immunofluorescence assays were conducted to evaluate the anti‐dengue virus (DENV) effects of these TCM compounds. Selected TCM formulas underwent testing for cytotoxicity and anti‐DENV activity. The study identified 62 promising TCM formulas, focusing on those most frequently prescribed and their potential efficacy. Among these, 23 TCM formulas showed promise in preventing severe illness, with 20 subjected to further analysis for cytotoxicity and anti‐DENV effects. Notably, Gastrodiae Rhizoma and Pinellia Rhizoma exhibited significant anti‐DENV activity at different multiplicities of infection (MOIs). This research introduces a novel methodology for identifying potential antiviral compounds against dengue fever, leveraging NHID data alongside immunofluorescence assays.

## Graphical abstract

Our study leverages Taiwan's NHI Database to identify and verify the antiviral properties of traditional Chinese medicine (TCM) against dengue fever, discovering that Gastrodia elata and Pinellia ternate exhibit significant anti‐DENV activity, positioning them as promising candidates for future antiviral drug development.

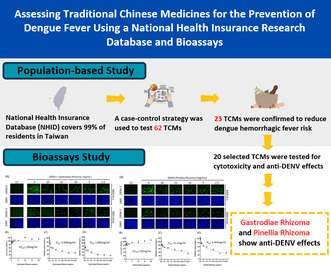

## 1 Introduction

Dengue fever (DF) is an infectious disease transmitted by mosquito vectors *Aedes aegypti* and *Aedes albopictus* mainly in tropical and subtropical regions. Roughly half of the globe faces the threat of DF, and it has been estimated that there are 390 million DF cases every year (WHO 2023). The *Aedes* mosquito in Taiwan has posed a persistent threat over the last several decades, including a pandemic involving roughly 60,000 cases in 2014 and 2015. The symptoms of DF typically include short‐term fever, pain, nausea, and vomiting; however, many individuals are susceptible to prolonged vomiting, abdominal pain, severe bleeding, and/or organ damage (WHO 2023). The dengue virus (DENV), the pathogen responsible for DF, enters cells by attaching to molecules or receptors on the host cell. Subsequent conformation changes in the envelope protein occur through endosome acidification, facilitating the release of the viral genome. The DENV genome includes three structural proteins—capsid (C), membrane (M), and envelope (E)—and seven non‐structural proteins: NS1, NS2A, NS2B, NS3, NS4A, NS4B, and NS5 (Nanaware et al. 2021). Note that the non‐structural proteins play a critical role in viral RNA genome replication and the cleavage of viral proteins. The C protein binds to the viral RNA genome, whereas the E and M proteins encapsulate the nuclear capsid core. After modification in the Golgi apparatus, the virus is released through budding during the final stage of its life cycle.

Vaccines have been developed for DF; however, they are not widely available. The treatment of dengue infection generally involves supportive or adjuvant therapies. Previous research has suggested that various Traditional Chinese medicine (TCM) formulas may enhance the anti‐DF effects of conventional treatments. In general, in the field of TCM, the treatment of DF is based on “wenbing” (febrile disease), which involves diagnosing and treating imbalances in the defensive qi, nutrient qi, and blood and the corresponding prescriptions are meant to clear heat and toxins via diuresis while cooling and nourishing the blood (Qin et al. 2023). Thus, relevant TCM treatments for heat‐clearing and detoxification may be useful for DF. In addition, one study, using hospital medical records, identified some TCM formulas, including Moutan Radicis Cortex, Isatidis Folium, Ophiopogonis Radix, Massa Medicata Fermentata, etc., frequently used in treating patients hospitalized with DF (Chen et al. 2020). Furthermore, some TCM candidates that appeared to inhibit the DENV in previous studies included Gastrodiae Rhizoma, Schisandrae Fructus, and Acori Tatarinowii Rhizoma (Qiu et al. 2007; Tong et al. 2010; Yao et al. 2018; Yu et al. 2017). However, here is currently very little clinical evidence that conclusively demonstrates the protective effects of relevant TCMs.

In this study, we used data from the National Health Insurance Database (NHID) in Taiwan to identify TCM formulas associated with a lower likelihood of developing dengue hemorrhagic fever (DHF). We also performed bioassays of the identified compounds to verify their antiviral effects against dengue viruses.

## 2 Materials and Methods

### 2.1 Screening for TCM Candidates Capable of Preventing DHF: A Population‐Based Case–Control Study

#### 2.1.1 Database

This case–control study utilized nationwide data sourced from the Longitudinal Health Insurance Database 2005 (LHID2005), which includes data from two million citizens randomly selected from the 2005 Registry of Beneficiaries (*n* = 25.68 million) of Taiwan's National Health Insurance (NHI) program. The NHI program, founded in 1995 and maintained by the Health and Welfare Data Science Center of the Taiwan Ministry of Health and Welfare, provides affordable and comprehensive medical services for more than 99% of all Taiwan residents. This means that individuals enlisted in LHID2005 accurately represent the entire populace of Taiwan. In the NHID, disease diagnoses were recorded using the International Classification of Diseases, Ninth Revision, Clinical Modification (ICD‐9‐CM) until 2016.

#### 2.1.2 Study Design and Sampled Participants

**FIGURE 1:**
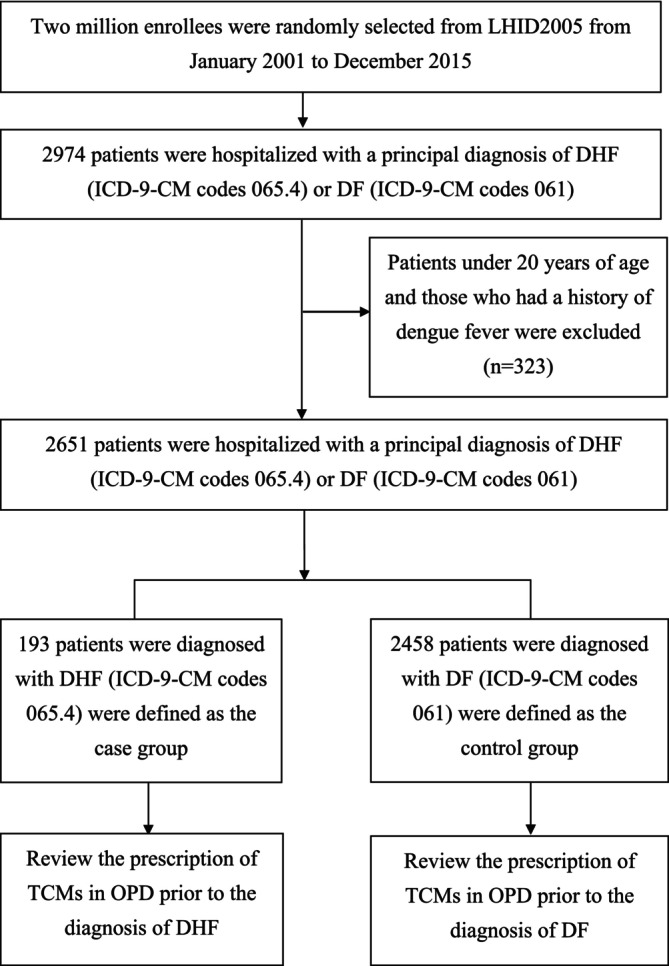
The flowchart of study design and sampled participants. DF, Dengue Fever; DHF, Dengue Hemorrhagic Fever; ICD, International Classification of Diseases; LHID2005, Longitudinal Health Insurance Database 2005; OPD, Outpatient Departments; TCMs, Conventional and Traditional Chinese Medicine.

This population‐based case–control study was designed to assess the association between exposure to specific TCM formulas and the risk of developing DHF among patients with DF. The primary aim was to identify TCM formulas with potential protective effects against DHF for further investigation. Initially, the study group contained 2974 patients who were hospitalized with a principal discharge diagnosis of DHF (ICD‐9‐CM codes 065.4) or DF (ICD‐9‐CM codes 061) from January 2001 to December 2015. Among these patients, 206 patients received a diagnosis of DHF and were defined as the case group. The remaining 2768 patients diagnosed with DF constituted the control group. To ensure that the study addressed only new cases of DF or DHF involving hospitalization since 2001, we excluded patients with a history of DF as of the year 2000 and those under 20 years of age to focus on the adult population (*n* = 323). Ultimately, 2651 patients were enrolled in the study, including 193 in the case group and 2458 in the control group (Figure 1).

#### 2.1.3 TCMs Candidates' Selection

The preliminary objective of this study was to identify TCM candidates based on their ability to prevent the onset of DHF among individuals with hemorrhagic fever in Taiwan. Table S1 listed the Latin names and Chinese names for all TCMs candidates in our study. Regarding the TCMs candidates in our study, those TCMs for heat‐clearing and detoxification (Qin et al. 2023), TCMs commonly used in treating patients hospitalized for DF (Chen et al. 2020), have potential effect to inhibit the DENV in prior literature were selected for the drug candidates (Qiu et al. 2007; Tong et al. 2010; Yao et al. 2018; Yu et al. 2017). However, we excluded rarely used TCM formulas in Taiwan to obtain dependable data in this study (LHID2005 regulations restrict the analysis of data with fewer than three observations to protect individual privacy and ensure data confidentiality, making it difficult to obtain reliable data.).

#### 2.1.4 Statistical Analysis

This study included data covering the period from January 2001 to December 2015. Chi‐squared tests were used to compare DF and DHF inpatients in terms of gender, region (southern and non‐southern areas), and the presence of comorbidities. An independent *t*‐test was performed to assess the divergence in age between case and control groups. Crude odds ratios (OR) were calculated via logistic regression, and the adjusted odds ratios (aOR) were obtained after adjusting for gender, age, region, year of admission, and comorbidities (such as chronic obstructive pulmonary disease, chronic kidney disease, coronary artery disease, gastrointestinal bleeding, hepatitis, diabetes, hypertension, hyperlipidemia, and ischemic stroke). The inverse probability of treatment weighting (IPTW) was adopted to mitigate the influence of confounding factors and selection bias by balancing the distribution of the various factors within the population of the case and control groups. A *p* value of 0.05 was adopted to indicate the level of significance. The SAS statistical package for Windows (version 9.4) was used for all statistical analysis.

### 2.2 Bioassays

#### 2.2.1 Cell Line and Virus

The culture conditions used for Vero cells (ATCC: CCL‐81) and DENV serotype 2 (DENV‐2, Strain: Thailand/16681/1984) were adopted from a previous study (Chen et al. 2021).

#### 2.2.2 TCMs

The TCM formulas assessed in this study were obtained from two TCM factories in Taiwan: Sun Ten pharmaceutical company (順天堂) and Sheng Chang pharmaceutical company (勝昌). Samples of each compound or formulation (10 g) were mixed into a 50% alcohol–water mixture (equivalent to 10× the quantity of the TCM) and allowed to stand at room temperature for at least 1 h. This mixture was filtered and then subjected to centrifugation at 25,000 g at 10°C for 30 mins, after which the supernatant was collected and concentrated at 45°C under rotation (50 rpm). The residual non‐volatile concentrate was dried at 40°C for at least 1 day, after which ultrapure water (500 μL) was gradually added. The dissolved concentrate extract was filtered through a 0.22 μm sterile syringe filter to obtain the final TCM stock.

#### 2.2.3 Cell Viability Assays

Cell viability was assessed using crystal violet assays in accordance with the methods outlined in a previous study (Chen et al. 2021).

#### 2.2.4 Immunofluorescence Assay

To determine the stage in which the anti‐DENV effects occur, Vero cells were seeded in 96‐well plates overnight and incubated overnight. The cells were then exposed to DENV‐2 at an MOI of 0.1 or 0.01 for 1 h, after which the inoculum was replaced with fresh medium. The cells were treated with the TCMs at specified concentrations according to the following schedule: co‐treatment (during whole infection), pre‐treatment 1 day (at 1 day prior to infection), and pre‐treatment 1 h (at 1 h prior to infection). Two days post‐inoculation, the treated cells were fixed and subjected to immunofluorescence staining. Treated cells were fixed using acetone and methanol at a ratio of 1:1 for 15 mins. The primary and secondary antibodies used for immunostaining were respectively the anti‐flavivirus E protein (PL1‐365, 1:400) and goat anti‐mouse IgG‐DyLight488 (Gentex, GTX213110‐04, 1:1000), with an incubation period of 1 hour. Nuclear staining was performed by incubating the cells with DAPI for 30 mins. The area affected by DENV infection was analyzed using ImageJ software after normalizing the various groups with the DENV group. The IC_50_ values of virus infection were obtained using GraphPad Prism software (Lu et al. 2023).

#### 2.2.5 RNA Isolation and Reverse Transcription‐Quantitative Polymerase Chain Reaction (RT‐qPCR)

Total RNA was extracted from the tested cells using the RNA extraction reagent (EBL, MRE‐3200). RNA expression levels were quantified utilizing the QuantiTect SYBR Green RT‐PCR Kit (Qiagen, 204243). The RT‐qPCR analysis was conducted on a Mic qPCR Cycler (Bio Molecular Systems) following this protocol: RT at 50°C for 30 min, initial denaturation at 95°C for 15 min, followed by 40 cycles of denaturation at 95°C for 20 s, annealing at 55°C for 30 s, and extension at 72°C for 30 s. The housekeeping gene actin was used as the internal control. DENV‐2 and actin primer sequences were as follows: DENV‐2‐F: 5′‐CAATATGCTGAAACGCGAGAGAAA‐3′; DENV‐2‐R: 5′‐AAGACATTGATGGCTTTTGA‐3′; Actin‐F: 5′‐ATTGCCGACAGGATGCAGAA‐3′; Actin‐R: 5′‐GCTGATCCACATCTGCTGGAA‐3′ (Chen et al. 2021; Lu et al. 2023).

## 3 Results

### 3.1 Screening of Potential TCMs Based on the Prevention of DHF: A Population‐Based Case–Control Study

#### 3.1.1 Baseline Characteristics

This case–control study included 193 inpatients diagnosed with DHF and 2458 inpatients diagnosed with DF as a control group. Table 1 lists the distribution of demographic characteristics and comorbidities between cases and controls. Note that the prevalence of DF in the control group was higher in the southern region of the country (0.94%) than in other regions (0.05%). A similar pattern was also observed in the case group (0.96% vs. 0.036%). In terms of comorbidities, we observed no statistically significant differences between the two groups in the prevalence of any condition except hypertension (41.5% vs. 49.74%, *p* = 0.0048). Adjusting and weighting models were used to mitigate differences between groups.

**TABLE 1:** Demographic characteristics and comorbidities of inpatients with DF or DHF in Taiwan (*n* = 2651).

Variables	Inpatients with DF (control group) *n* = 2458		Inpatients with DHF (case group) *n* = 193		*p*
	*n*	%	*n*	%	
Gender					
Male	1251	50.9	93	48.19	0.9754
Female	1207	49.1	100	51.81	
Age (years)					0.0524
Region					
Other regions	125	5.09	7	3.63	0.9713
Southern region	2333	94.91	186	96.37	
Comorbidities					
Chronic obstructive pulmonary disease	508	20.67	38	19.69	0.9162
Chronic kidney disease	331	13.47	27	13.99	0.4413
Coronary artery disease	572	23.27	42	21.76	0.8017
Gastrointestinal bleeding	1750	71.20	115	59.59	0.9805
Hepatitis	234	9.52	14	7.25	0.7517
Diabetes	669	27.22	59	30.57	0.3758
Hypertension	1020	41.50	96	49.74	0.0048
Hyperlipidemia	896	36.45	62	32.12	0.9864
Ischemic stroke	372	15.13	24	12.44	0.2383

#### 3.1.2 Primary Outcomes

We selected 62 candidate TCM formulas based on their potential effects against DHF (Qiu et al. 2007; Tong et al. 2010; Yao et al. 2018; Yu et al. 2017). Table 2 presents the correlations between candidate TCM formulas and DHF. Significant protective effects were associated with the following TCM formulas: Acori Tatarinowii Rhizoma, Angelicae Dahuricae Radix, Bletillae Rhizoma, Gan‐Lu‐Yin, Ge‐Gen‐Tang, Glycyrrhizae Radix et Rhizome, Jhih‐Gan‐Cao‐Tang, Jing‐Fang‐Bai‐Du‐San, Lonicerae Japonicae Flos, Magnoliae Cortex, Massa Medicata Fermentata, Ophiopogonis Radix, Paeoniae Radix Alba, Pinella and Magnolia Combination, Puerariae Radix, Salviae Miltiorrhizae Radix et Rhizoma, Scutellariae Radix, and Siao‐Chai‐Hu‐Tang.

**TABLE 2:** The occurrence, crude odds ratio (OR), adjusted odds ratios (aOR), inverse probability of treatment weighting odds ratios (IPTW‐OR), and 95% confidence intervals (CIs) between the candidate TCMs and DHF among dengue fever inpatients.

TCMs exposure	Inpatients with DF (*n* = 2458)		Inpatients with DHF (*n* = 193)		Crude OR (95% CI)	Adjusted OR (95% CI)	IPTW‐OR (95% CI)
	*n*	%	*n*	%			
Acori Tatarinowii Rhizoma	193	7.85	4	2.07	0.248** (0.091–0.676)	0.431*** (0.331–0.560)	0.446*** (0.341–0.584)
Angelicae Dahuricae Radix	386	15.7	17	8.81	0.518* (0.312–0.863)	0.606*** (0.511–0.720)	0.647*** (0.540–0.775)
Bletillae Rhizoma	163	6.63	4	2.07	0.298* (0.109–0.812)	0.484*** (0.368–0.637)	0.535*** (0.403–0.710)
Bupleuri Radix	207	8.42	19	9.84	1.187 (0.724–1.947)	1.983*** (1.654–2.377)	2.283*** (1.887–2.761)
Cassiae Semen	78	3.17	3	1.55	0.482 (0.151–1.541)	0.997 (0.722–1.377)	1.056 (0.758–1.472)
Chai Ge Jie Ji Tang	178	7.24	7	3.63	0.482 (0.223–1.041)	0.527*** (0.408–0.681)	0.554*** (0.427–0.720)
Coptidis Rhizoma	241	9.8	13	6.74	0.664 (0.373–1.185)	0.861 (0.707–1.047)	0.933 (0.759–1.147)
Forsythiae Fructus	266	10.82	14	7.25	0.645 (0.369–1.127)	1.035 (0.864–1.241)	1.1 (0.910–1.331)
Gan‐Lu‐Siao‐Du‐Dan	147	5.98	8	4.15	0.68 (0.329–1.407)	1.027 (0.810–1.302)	1.117 (0.872–1.430)
Gan‐Lu‐Yin	350	14.24	11	5.7	0.364** (0.196–0.676)	0.638*** (0.534–0.761)	0.647*** (0.537–0.778)
Gardeniae Fructus	199	8.1	11	5.7	0.686 (0.367–1.283)	0.576*** (0.454–0.730)	0.644** (0.503–0.823)
Gastrodiae Rhizoma	182	7.4	7	3.63	0.471 (0.218–1.016)	0.425*** (0.324–0.558)	0.468*** (0.354–0.619)
Ge‐Gen‐Tang	503	20.46	26	13.47	0.605* (0.396–0.925)	0.653*** (0.562–0.759)	0.714*** (0.610–0.837)
Glycyrrhizae Radix et Rhizoma Praeparatum cum Melle	44	1.79	4	2.07	1.161 (0.413–3.266)	1.353 (0.622–2.943)	1.379 (0.582–3.267)
Glycyrrhizae Radix et Rhizome	448	18.23	21	10.88	0.548* (0.344–0.872)	0.1*** (0.051–0.197)	0.084*** (0.042–0.171)
Gypsum Fibrosum	64	2.6	5	2.59	0.995 (0.396–2.502)	1.888*** (1.380–2.583)	2.137*** (1.539–2.966)
Huang‐Cin‐Huang‐Lian‐Tang	56	2.28	3	1.55	0.678 (0.210–2.185)	0.162* (0.033–0.792)	0.17* (0.031–0.923)
Huo‐Siang‐Jheng‐Ci‐San	269	10.94	15	7.77	0.686 (0.399–1.179)	0.881 (0.732–1.061)	0.89 (0.731–1.082)
Isatidis Folium	62	2.52	6	3.11	1.24 (0.530–2.905)	4.104*** (3.076–5.476)	4.454*** (3.272–6.062)
Isatidis Radix	172	7	8	4.15	0.575 (0.278–1.186)	0.868 (0.690–1.091)	0.953 (0.748–1.214)
Jhih‐Gan‐Cao‐Tang	256	10.41	10	5.18	0.47* (0.246–0.900)	1.117 (0.778–1.603)	1.223 (0.797–1.876)
Jing‐Fang‐Bai‐Du‐San	290	11.8	9	4.66	0.366** (0.185–0.722)	0.32*** (0.253–0.406)	0.32*** (0.251–0.407)
Lonicerae Japonicae Flos	205	8.34	7	3.63	0.414* (0.192–0.892)	0.695** (0.556–0.868)	0.783* (0.618–0.991)
Magnoliae Cortex	307	12.49	14	7.25	0.548* (0.314–0.956)	0.842 (0.706–1.005)	0.858 (0.712–1.034)
Massa Medicata Fermentata	196	7.97	5	2.59	0.307* (0.125–0.755)	0.343*** (0.259–0.455)	0.338*** (0.254–0.450)
Moutan Radicis Cortex	177	7.2	16	8.29	1.165 (0.683–1.987)	1.483*** (1.21–1.817)	1.763*** (1.422–2.185)
Notopterygii Rhizoma et Radix	228	9.28	12	6.22	0.648 (0.356–1.181)	0.955 (0.784–1.162)	1.038 (0.845–1.275)
Notopterygium Nine Herb Combination	74	3.01	4	2.07	0.683 (0.247–1.886)	2.416** (1.29–4.527)	3.101** (1.557–6.177)
Ophiopogonis Radix	330	13.43	15	7.77	0.543* (0.317–0.932)	0.732*** (0.614–0.873)	0.766** (0.637–0.921)
*Paeonia Lactiflora*	85	3.46	9	4.66	1.366 (0.676–2.760)	0.226** (0.077–0.664)	0.291* (0.096–0.879)
Paeoniae Radix Alba	239	9.72	5	2.59	0.247** (0.101–0.606)	0.403*** (0.317–0.514)	0.388*** (0.303–0.498)
Pinella and Gastrodia Combination	162	6.59	9	4.66	0.693 (0.348–1.379)	0.883 (0.698–1.116)	0.9 (0.700–1.157)
Pinella and Magnolia Combination	497	20.22	25	12.95	0.587* (0.381–0.904)	0.612*** (0.525–0.714)	0.669*** (0.570–0.785)
Pinellia rhizoma	225	9.15	10	5.18	0.542 (0.283–1.040)	0.706** (0.571–0.872)	0.720** (0.576–0.899)
Platycodonis Radix	465	18.92	26	13.47	0.667 (0.436–1.021)	1.01 (0.875–1.167)	1.145 (0.980–1.338)
Puerariae Radix	450	18.31	19	9.84	0.487** (0.300–0.791)	0.708*** (0.606–0.828)	0.749** (0.635–0.883)
Qiang Huo Sheng Shi Tang	132	5.37	6	3.11	0.565 (0.246–1.299)	0.531* (0.291–0.972)	0.5* (0.259–0.966)
Salviae Miltiorrhizae Radix et Rhizoma	401	16.31	19	9.84	0.56* (0.345–0.910)	0.602*** (0.508–0.713)	0.61*** (0.510–0.730)
Schisandrae Fructus	210	8.54	11	5.7	0.647 (0.346–1.208)	0.792* (0.641–0.98)	0.825 (0.661–1.031)
Schizonepeta and Forsythia Combination	117	4.76	4	2.07	0.424 (0.155–1.160)	0.722* (0.542–0.963)	0.674* (0.497–0.913)
Scutellariae Radix	447	18.19	19	9.84	0.491** (0.303–0.798)	0.642*** (0.548–0.753)	0.669*** (0.566–0.792)
Siao‐Chai‐Hu‐Tang	383	15.58	17	8.81	0.523* (0.314–0.871)	1.055 (0.739–1.505)	0.981 (0.645–1.493)
Tian‐Ma‐Gou‐Teng‐Yin	119	4.84	9	4.66	0.962 (0.481–1.925)	0.969 (0.743–1.264)	1.209 (0.913–1.603)
Yin‐Ciao‐San	438	17.82	29	15.03	0.816 (0.542–1.227)	1.1 (0.960–1.283)	1.203* (1.033–1.400)
Zhu Ye Shi Gao Tang	99	4.03	6	3.11	0.765 (0.331–1.767)	1.166 (0.884–1.538)	1.224 (0.920–1.627)

* *p* < 0.05.

** *p* < 0.01.

*** *p* < 0.001.

After adjusting for baseline characteristics, some of the TCMs demonstrated protective effects in both the case and control groups. TCMs with a notable protective effect in associated the risk of DHF were as follows: Acori Tatarinowii Rhizoma, Angelicae Dahuricae Radix, Bletillae Rhizoma, Chai Ge Jie Ji Tang, Gan‐Lu‐Yin, Gardeniae Fructus, Gastrodiae Rhizoma, Ge‐Gen‐Tang, Glycyrrhizae Radix et Rhizome, Huang‐Cin‐Huang‐Lian‐Tang, Jing‐Fang‐Bai‐Du‐San, Lonicerae Japonicae Flos, Massa Medicata Fermentata, Ophiopogonis Radix, *Paeonia Lactiflora* , Paeoniae Radix Alba, Pinella and Magnolia Combination, Pinellia rhizoma, Puerariae Radix, Qiang Huo Sheng Shi Tang, Salviae Miltiorrhizae Radix et Rhizoma, Schisandrae Fructus, Schizonepeta and Forsythia Combination, and Scutellariae Radix.

This study also used the IPTW method to alter baseline imbalances. The following TCMs were shown to significantly reduced the risk of DHF in IPTW analysis: Acori Tatarinowii Rhizoma, Angelicae Dahuricae Radix, Bletillae Rhizoma, Chai Ge Jie Ji Tang, Gan‐Lu‐Yin, Gardeniae Fructus, Gastrodiae Rhizoma, Ge‐Gen‐Tang, Glycyrrhizae Radix et Rhizome, Huang‐Cin‐Huang‐Lian‐Tang, Jing‐Fang‐Bai‐Du‐San, Lonicerae Japonicae Flos, Massa Medicata Fermentata, Ophiopogonis Radix, *Paeonia Lactiflora* , Paeoniae Radix Alba, Pinella and Magnolia Combination, Pinellia rhizoma, Puerariae Radix, Qiang Huo Sheng Shi Tang, Salviae Miltiorrhizae Radix et Rhizoma, Schizonepeta and Forsythia Combination, and Scutellariae Radix.

In brief summary, our study initially selected 62 candidate TCM formulas based on their potential effects against DHF in prior literature. The population‐based case–control study further revealed that 29 formulas were significantly associated with DHF onset. Among these, 23 formulas, consisting of 15 individual compounds and eight combinations, demonstrated a significant “protective effect” in the IPTW‐OR analysis. We selected these 15 individual compounds, along with five others that showed no protective effect in population‐based study (as negative controls), for further bioassays.

### 3.2 *Gastrodia elata* ( *G. elata* ) and *Pinellia ternate* ( *P. ternate* ) Presented Anti‐DENV Effects

**FIGURE 2:**
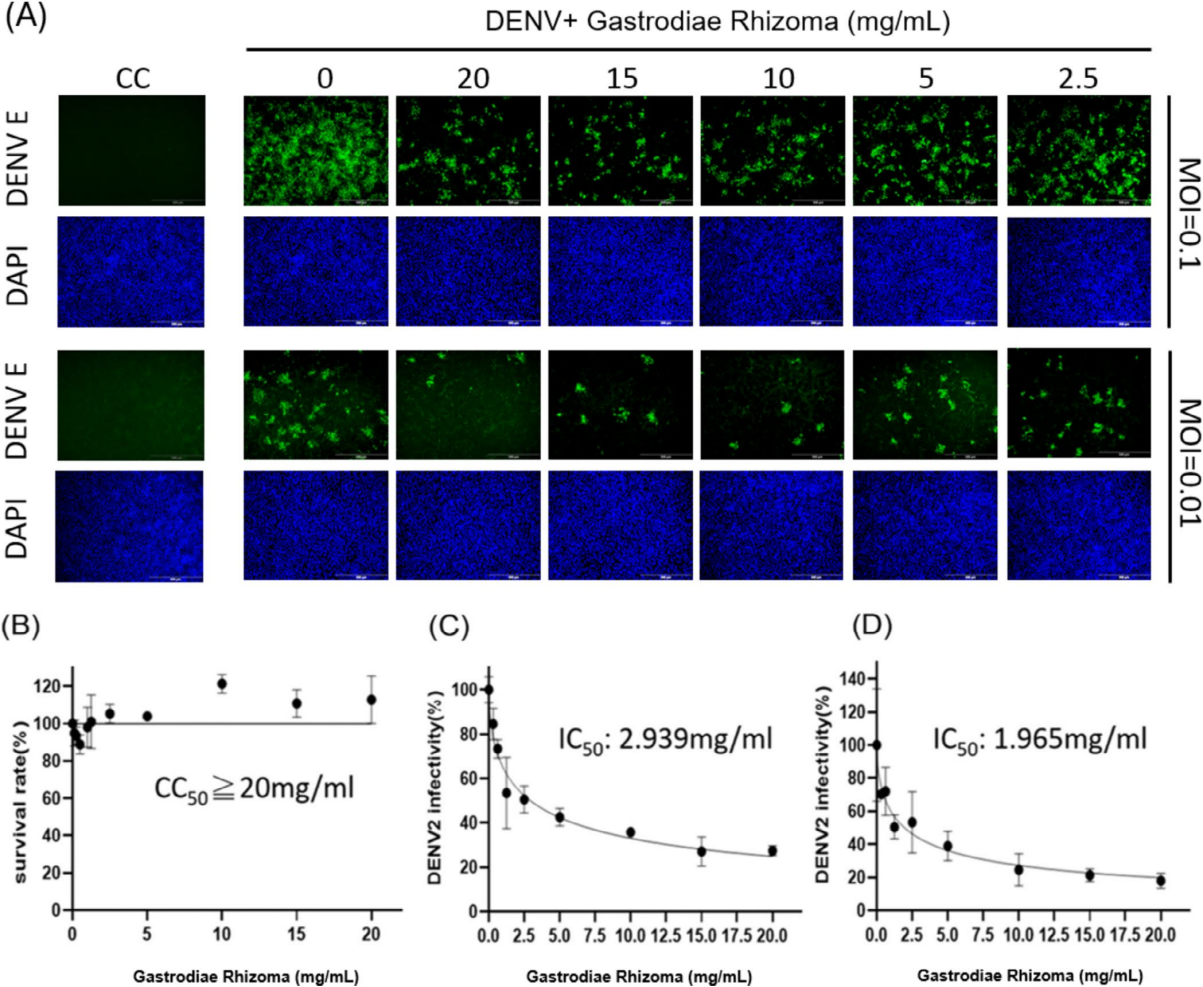
(A) Effects of Gastrodiae Rhizoma on the proliferation of Vero cell when co‐administered with DENV‐2 at an MOI of 0.1 or 0.01, where green fluorescence indicates DENV envelop protein and DAPI indicates staining of the cell nucleus; (B) Crystal violet assay results indicating cell survival rate; DENV2 infectivity and IC_50_ of Gastrodiae Rhizoma at MOI = 0.1 (C) and MOI = 0.01 (D) were calculated as a percentage based on IFA using ImageJ software. Scale bar: 200 μm.

**FIGURE 3:**
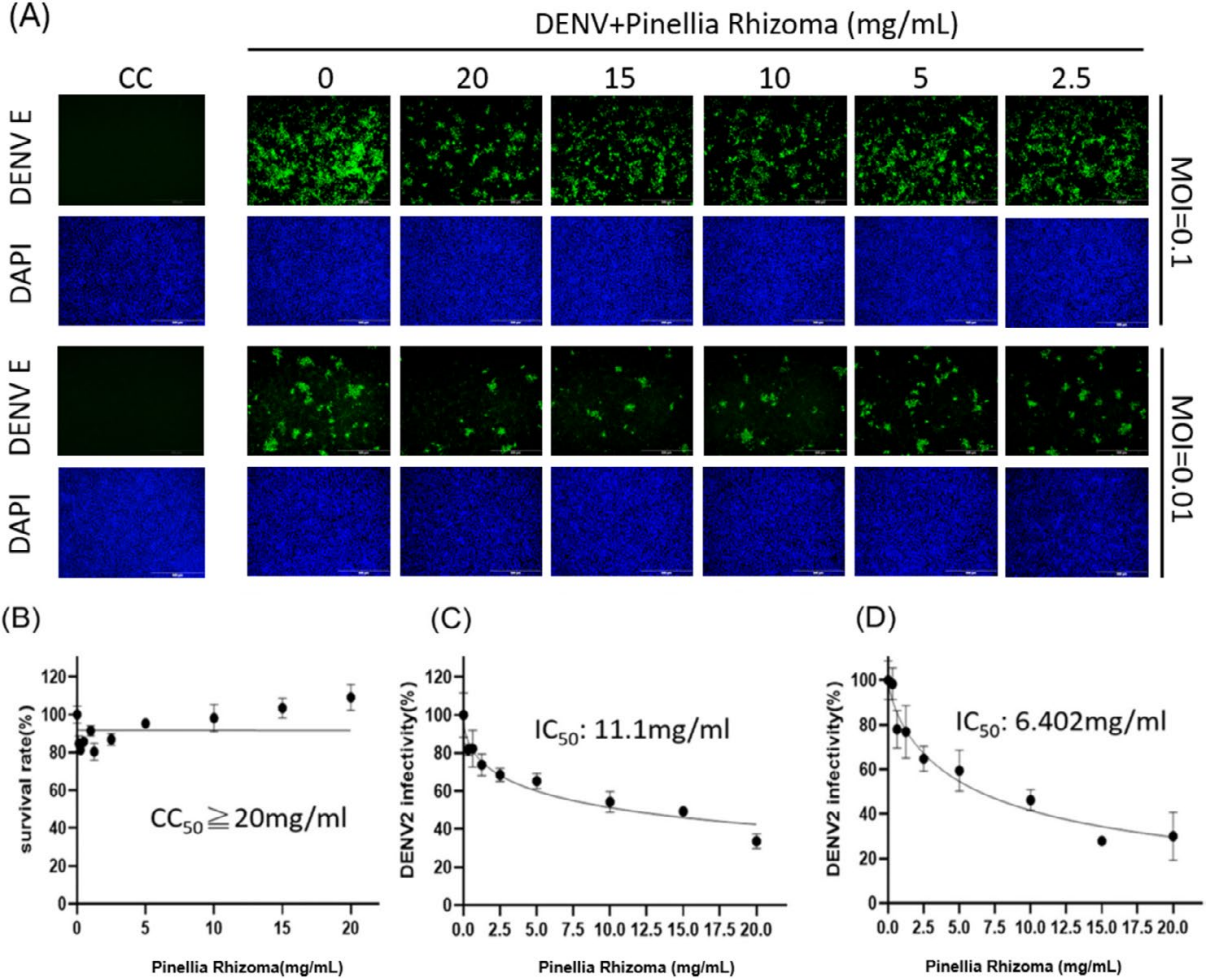
(A) Effects of Pinellia Rhizoma on the proliferation of Vero cell when co‐administered with DENV‐2 at an MOI of 0.1 or 0.01, where green fluorescence indicates DENV envelop protein and DAPI indicates staining of the cell nucleus; (B) Crystal violet assay results indicating cell survival rate; DENV2 infectivity and IC_50_ of Pinellia Rhizoma at MOI = 0.1 (C) and MOI = 0.01 (D) were calculated as a percentage based on IFA using ImageJ software. Scale bar: 200 μm.

The preliminary findings revealed 20 TCM candidates for bioassay. The safety of the extracts was assessed via crystal violet assay to determine cell viability after TCM treatment for 2 days. CC_50_ values exceeding 20 mg/mL were observed in five candidates: Gastrodiae Rhizoma, Pinellia Rhizoma, Massa Medicata Fermentata, Angelicae Dahuricae Radix, and Gardeniae Fructus. Table 3 lists the CC_50_ and IC_50_ values of the 20 TCMs in co‐treatment group, pre‐treatment 1 day group, and pre‐treatment 1 h group. As shown in Figures 2 and 3, only Gastrodiae Rhizoma and Pinellia Rhizoma presented anti‐DENV‐2 effects during the co‐treatment group. The IC_50_ values at an MOI = 0.1 were as follows: Gastrodiae Rhizoma (2.94 mg/mL) and Pinellia Rhizoma (11.1 mg/mL). The IC_50_ values at an MOI = 0.01 were as follows: Gastrodiae Rhizoma (1.965 mg/mL) and Pinellia Rhizoma (6.4 mg/mL). These immunofluorescence staining results were further validated using RT‐qPCR, as shown in Figure S1.

**TABLE 3:** Cell viability analysis (CC_50_) and IFA staining results (IC_50_) of 20 TCMs in different experimental designs.

TCMs	Cell viability	Co‐treatment	Pre‐treatment 1 day	Pre‐treatment 1 h
	CC_50_ (mg/mLL)	IC_50_ (mg/mL)	IC_50_ (mg/mL)	IC_50_ (mg/mL)
Gastrodiae Rhizoma	≧ 20	2.94 (MOI = 0.1)	—	—
		1.97 (MOI = 0.01)		
		– (MOI = 1)		
Pinellia Rhizoma	≧ 20	11.1 (MOI = 0.1)	—	—
		6.4 (MOI = 0.01)		
		– (MOI = 1)		
Massa Medicata Fermentata	≧ 20	—	—	—
Bletillae Rhizoma	12.9	—	—	—
Angelicae Dahuricae Radix	≧ 20	—	—	—
Gardeniae Fructus	6.75	—	—	—
Ophiopogonis Radix	≧ 10	—		
Scutellariae Radix	0.33	—	—	—
Puerariae Radix	≧ 10	—	—	—
Salviae Miltiorrhizae Radix et Rhizoma	1.78	—	—	—
Glycyrrhizae Radix et Rhizome	0.27	—	—	—
Paeoniae Radix Alba	0.42	—	—	—
Acori Tatarinowii Rhizoma	7.33	—	—	—
Lonicerae Japonicae Flos	5.39	—	—	—
^a^ Isatidis Folium	3.95	—	—	—
^a^ Moutan Radicis Cortex	0.706	—	—	—
^a^ Bupleuri Radix	7.52	—	—	—
^a^ Platycodonis Radix	0.9716	—	—	—
^a^ Glycyrrhizae Radix et Rhizoma Praeparatum cum Melle	1.06	—	—	—
Forsythiae Fructus	0.6	—	—	—

*Note:* –: The drug did not suppress viral infection under co‐treatment or the drug did not prevent viral infection under 1 day pre‐treatment or 1 h pre‐treatment.

^a^ Negative control in this study.

## 4 Discussion

TCM treatments are commonly used in Taiwan for the prevention and treatment of disease. The fact that the Taiwan FDA has approved the sale of numerous TCM products and that the NHID pays for many TCM products makes it possible to assess the benefits of TCM remedies in preventing/treating DENV infection. It is well‐established that DENV infection can lead to dengue disease. Although most individuals remain asymptomatic, approximately one‐fourth may develop DF. Clinical symptoms typically include fever, headache, retro‐orbital pain, joint pains, weakness, and rash. Some individuals may progress to severe dengue, such as DHF or dengue hemorrhagic shock, particularly following a secondary infection. Patients with severe dengue may experience severe abdominal pain, persistent vomiting, bleeding, fatigue, and the presence of blood in vomit or stool. Our study adopts a novel approach by integrating a population‐based case–control study with subsequent bioassays to explore the antiviral effects of TCM against DENV. This method is innovative because it combines real‐world epidemiological evidence with mechanistic validation, bridging the gap between clinical observation and biological mechanisms. Unlike traditional approaches that often rely on expert opinions or literature reviews, we used large‐scale population data to identify TCM formulas associated with a lower likelihood of developing DHF. This evidence‐based approach provides a robust and data‐driven foundation for selecting candidate formulas, contributing to a deeper understanding of alternative strategies for DHF prevention. TCMs that may protect against DHF could have antiviral effects. Previous studies have shown that DHF patients have significantly higher viral loads and greater immune activity compared with DF patients (W. K. Wang et al. 2003, 2006). It is suggested that reducing the viral load could make it less likely for patients to develop severe symptoms. Therefore, drugs that offer protection against DHF may work by suppressing the virus. This article aims to highlight that by using health‐care database strategies, we may be able to identify promising antiviral drugs. Note that this analysis was meant to assess the effects of these drugs in inhibiting viral replication, inducing host antiviral mechanisms, and disrupting binding between the virus and host receptors.

The complex pathogenesis of DHF is largely determined by viral binding to specific antibodies, which can induce a series of disorders related to blood clotting (Azeredo, Monteiro, and De‐Oliveira Pinto 2015; Bournazos, Gupta, and Ravetch 2020; Halstead 1988). The fact that Gastrodiae Rhizoma and Pinellia Rhizoma were shown to protect against DHF as well as inhibit DENV infection suggests that they interfere with the process of DENV infection. It is important to consider that these drugs did not demonstrate preventive effects against DENV, the testing of which may require animal models. It is also important to consider the complexity of the active ingredients in these compounds and the degree to which the manufacturing process could affect virus–cell interactions.

Qiu et al. (2007) determined that two structural glucans (WGEW and AGEW) and the corresponding sulfide derivatives isolated from Gastrodiae Rhizoma inhibited the replication of dengue viruses. They posited that sulfide derivatives prevented early‐stage viral infection by interfering with viral adsorption and penetration (Tong et al. 2010). We were unable to find any previous report on the effects of Pinellia Rhizoma against DENV. Researchers have previously reported that Pinellic acid in Pinellia Rhizoma could be used as a co‐adjuvant to promote the expression of antiviral IgA antibodies (X. Wang et al. 2006). It has also been suggested that Pinellia Rhizoma extract could be used to inhibit Ebola virus infection (Yang et al. 2017).

This research has several limitations. In our population‐based study, we employed case–control design, which is commonly used to investigate multiple exposures, such as TCMs, within in study population. However, this design may introduce potential biases. In bioassay, we implemented three treatments treatment protocols: co‐treatment, pre‐treatment 1 day, prior to infection, and pre‐treatment 1 h prior to infection, to evaluate the antiviral effects and the host antiviral interaction of TCMs. However, determining whether TCMs are beneficial for preventing or treating DHF is challenging due to the complex nature of the disease progression. Some individuals may progress into DHF during a secondary infection. Research indicates that antibody‐dependent enhancement (ADE) might facilitate viral infection. Additionally, DHF can result from factors such as coagulation and fibrinolysis influenced by cytokines during DENV infection, inhibition of prothrombin activation by the DENV NS1 protein, and autoantibody production resulting from molecular mimicry of DENV (Chuang et al. 2013). Actually, we initially expected to observe effects in the pre‐treatment 1‐day group. However, we found that only Gastrodiae Rhizoma and Pinellia Rhizoma exhibited anti‐DENV effects in the co‐treatment group. This finding encourages us to explore the potential of this strategy for screening other mechanism‐based antiviral drugs.

## 5 Conclusions

In this study, we adopted a novel approach to the identification of antiviral candidates through the use of NHID data followed by bioassays. This study design could also be used to assess the long‐term benefits of antiviral medications. Our analysis revealed two TCMs (Gastrodiae Rhizoma and Pinellia Rhizoma) with significant antiviral effects worthy of further research.

## Author Contributions

**Wan‐Yu Su:** conceptualization (equal), data curation (equal), formal analysis (equal), funding acquisition (equal), investigation (equal), methodology (equal), project administration (equal), resources (equal), software (equal), supervision (equal), validation (equal), visualization (equal), writing – original draft (equal), writing – review and editing (equal). **Hsiang‐Hui Lo:** conceptualization (equal), data curation (equal), formal analysis (equal), funding acquisition (equal), investigation (equal), methodology (equal), project administration (equal), resources (equal), software (equal), supervision (equal), validation (equal), visualization (equal), writing – original draft (equal), writing – review and editing (equal). **Yuan‐Liang Wen:** conceptualization (equal), supervision (equal), validation (equal), writing – original draft (equal), writing – review and editing (equal). **Jeng‐Wei Lu:** methodology (equal), supervision (equal), visualization (equal), writing – original draft (equal), writing – review and editing (equal). **I‐Chuan Yen:** supervision (equal), validation (equal), visualization (equal), writing – original draft (equal), writing – review and editing (equal). **Yi‐Jung Ho:** conceptualization (equal), data curation (equal), formal analysis (equal), funding acquisition (equal), investigation (equal), methodology (equal), project administration (equal), resources (equal), software (equal), supervision (equal), validation (equal), visualization (equal), writing – original draft (equal), writing – review and editing (equal). **Li‐Ting Kao:** conceptualization (equal), data curation (equal), formal analysis (equal), funding acquisition (equal), investigation (equal), methodology (equal), project administration (equal), resources (equal), software (equal), supervision (equal), validation (equal), visualization (equal), writing – original draft (equal), writing – review and editing (equal).

## Conflicts of Interest

L.T.‐K. reports research funding outside the submitted work from IQVIA. The other authors declare no conflicts of interest.

## Supporting information


**Figure S1.** Reverse transcription‐quantitative polymerase chain reaction (RT‐qPCR) results (normalized using actin) showing DENV‐2 RNA levels following *G. elata* and *P. ternata* treatment. Quantification data in (A) and (B) were obtained from at least three independent experiments. Significance is indicated as follows: *p < 0.05; **p < 0.01.

**Table S1.** Latin name (or English name) and Chinese name for Traditional Chinese Medicines.

## Data Availability

Data used in this study are handled and stored by the Health and Welfare Data Science Center. Interested researchers can obtain the data through formal application to the Health and Welfare Data Science Center, Department of Statistics, Ministry of Health and Welfare, Taiwan.
