# Supplementary material for: Assessing Traditional Chinese Medicines for Anti‐Dengue Using a National Health Insurance Research Database and Bioassays
Source: Food Sci Nutr. 2025 Feb 28;13(3):e70009. doi: 10.1002/fsn3.70009 (PMC11868784; doi:10.1002/fsn3.70009)
Supplement: Supplementary file 1 — Figure S1. Reverse transcription‐quantitative polymerase chain reaction (RT‐qPCR) results (normalized using actin) showing DENV‐2 RNA levels following G. elata and P. ternata treatment. Quantification data in (A) and (B) were obtained from at least three independent experiments. Significance is indicated as follows: *p < 0.05; **p < 0.01. [file FSN3-13-e70009-s001.docx]

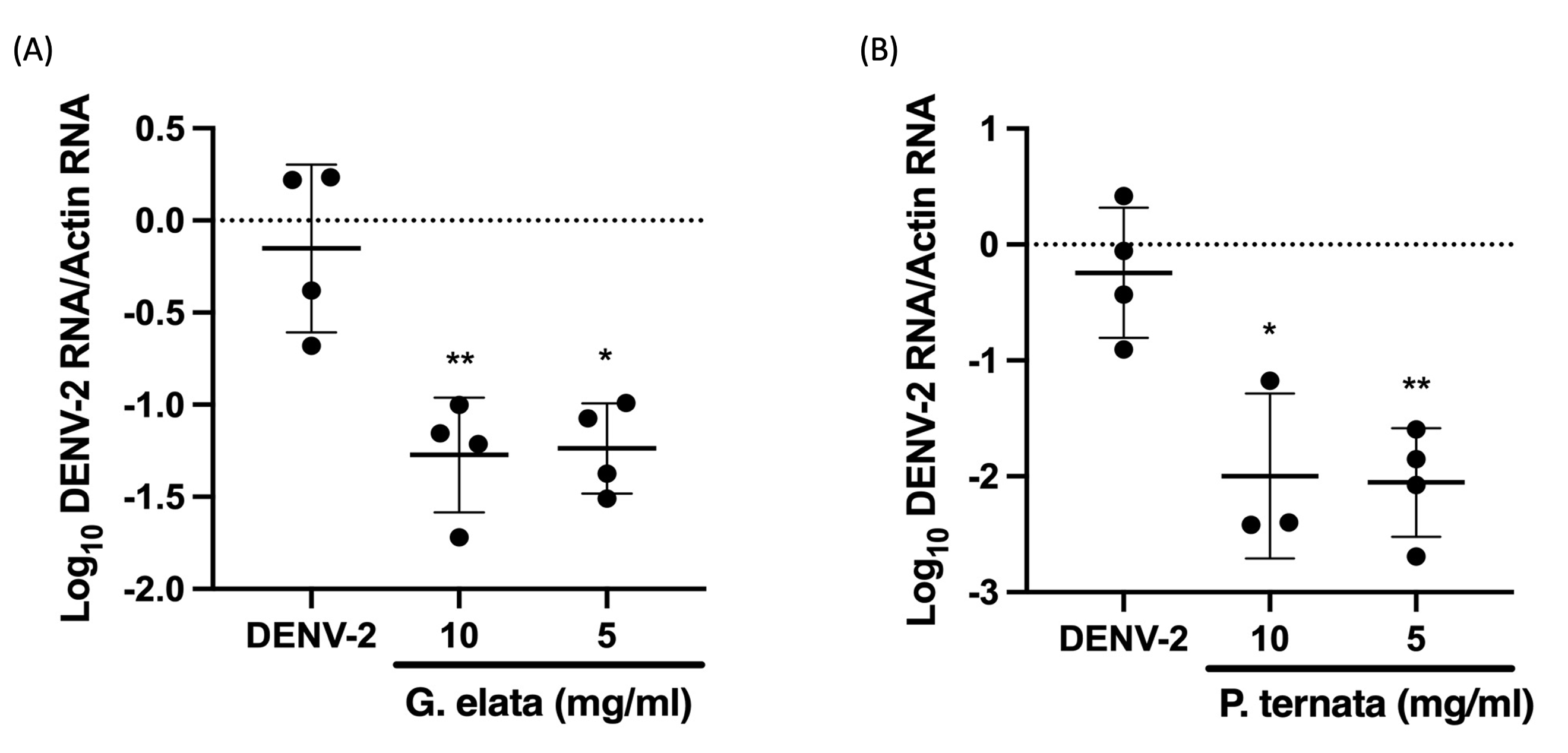


**Supplementary Fig. 1.** Reverse transcription‐quantitative polymerase chain reaction (RT-qPCR) results (normalized using actin) showing DENV‐2 RNA levels following G. elata and P. ternata treatment. Quantification data in (A) and (B) were obtained from at least three independent experiments. Significance is indicated as follows: *p<0.05; **p<0.01.
