# Supplementary material for: Assessing Traditional Chinese Medicines for Anti‐Dengue Using a National Health Insurance Research Database and Bioassays
Source: Food Sci Nutr. 2025 Feb 28;13(3):e70009. doi: 10.1002/fsn3.70009 (PMC11868784; doi:10.1002/fsn3.70009)
Supplement: Supplementary file 2 — Table S1. Latin name (or English name) and Chinese name for Traditional Chinese Medicines. [file FSN3-13-e70009-s002.docx]

**Supplementary Table1**. Latin name (or English name) and Chinese name for Traditional Chinese Medicines.

| **Latin name or English name** | **Chinese name** |
| --- | --- |
| Acori Tatarinowii Rhizoma | 石菖蒲 |
| Agrimoniae Herba | 仙鶴草 |
| Angelicae Dahuricae Radix | 白芷 |
| Asini Corii Colla | 阿膠 |
| Bletillae Rhizoma | 白及 |
| Bupleuri Radix | 柴胡 |
| Cassiae Semen | 決明子 |
| Chai Ge Jie Ji Tang | 柴葛解肌湯 |
| Coptidis Rhizoma | 黃連 |
| Da-Chai-Hu-Tang | 大柴胡湯 |
| Fei Er Pills | 肥兒丸 |
| Forsythia Detoxification Powder | 連翹敗毒散 |
| Forsythiae Fructus | 連翹 |
| Galli Gigerii Corneum Endothelium | 雞內金 |
| Gan-Lu-Siao-Du-Dan | 甘露消毒丹 |
| Gan-Lu-Yin | 甘露飲 |
| Gardenia And Fermented Soybean Decoction | 梔子豉湯 |
| Gardenia Liver-Cleansing Decoction | 梔子清肝湯 |
| Gardeniae Fructus | 梔子 |
| Gastrodiae Rhizoma | 天麻 |
| Ge-Gen-Tang | 葛根湯 |
| Glycyrrhizae Radix et Rhizoma Praeparatum Cum Melle | 甘草和炙甘草 |
| Glycyrrhizae Radix et Rhizoma Praeparatum Cum Melle | 炙甘草 |
| Glycyrrhizae Radix et Rhizome | 甘草 |
| Guei-Pi-Tang | 歸脾湯 |
| Gypsum Fibrosum | 石膏 |
| Gypsum Fibrosum And Mentha Combination | 清瘟敗毒飲 |
| Huang-Cin-Huang-Lian-Tang | 黃芩黃連湯 |
| Huang-Lian-Jie-Du-Tang | 黃連解毒湯 |
| Huang-Lian-Tang | 黃連湯 |
| Huo-Siang-Jheng-Ci-San | 藿香正氣散 |
| Isatidis Folium | 大青葉 |
| Isatidis Radix | 北板藍根 |
| Jhih-Gan-Cao-Tang | 炙甘草湯 |
| Jing-Fang-Bai-Du-San | 荊防敗毒散 |
| Lonicerae Japonicae Flos | 金銀花 |

**Supplementary Table1**. Latin name (or English name) and Chinese name for Traditional Chinese Medicines. (continued)

| **Latin name or English name** | **Chinese name** |
| --- | --- |
| Magnoliae Cortex | 厚朴 |
| Massa Medicata Fermentata | 神麴 |
| Moutan Radicis Cortex | 牡丹皮 |
| Notopterygii Rhizoma Et Radix | 羌活 |
| Notopterygium Nine Herb Combination | 九味羌活湯 |
| Ophiopogonis Radix | 麥門冬 |
| Paeoniae Radix Alba | 白芍 |
| Pinella And Gastrodia Combination | 半夏白朮天麻湯. |
| Pinella And Magnolia Combination | 半夏厚朴湯 |
| Pinellia Rhizoma | 半夏 |
| Platycodon Combination | 桔梗湯 |
| Platycodonis Radix | 桔梗 |
| Puerariae Radix | 葛根 |
| Qiang Huo Sheng Shi Tang | 羌活勝濕湯 |
| Qing Shu Yi Qi Tang | 清暑益氣湯 |
| Salviae Miltiorrhizae Radix et Rhizoma | 丹參 |
| Schisandrae Fructus | 五味子 |
| Schizonepeta And Forsythia Combination | 荊芥連翹湯 |
| Scutellariae Radix | 黃芩 |
| Shao Yao Tang | 芍藥湯 |
| Shao-Yao-Gan-Cao-Tang | 芍藥甘草湯 |
| Siao-Chai-Hu-Tang | 小柴胡湯 |
| Tian-Ma-Gou-Teng-Yin | 天麻鉤藤飲 |
| Triple Nut Decoction | 三仁湯 |
| Yin-Ciao-San | 銀翹散 |
| Zhu Ye Shi Gao Tang | 竹葉石膏湯 |
